# Identification of an Endoplasmic Reticulum Stress-Related Gene Signature to Evaluate the Immune Status and Predict the Prognosis of Hepatocellular Carcinoma

**DOI:** 10.3389/fgene.2022.850200

**Published:** 2022-05-27

**Authors:** Dingli Song, Zhenyu Zhou, Dai Zhang, Jie Wu, Qian Hao, Lili Zhao, Hong Ren, Boxiang Zhang

**Affiliations:** ^1^ Department of Thoracic Surgery, The First Affiliated Hospital of Xi’an Jiaotong University, Xi’an, China; ^2^ Department of Hepatobiliary Surgery, Sun Yat-Sen Memorial Hospital, Sun Yat-Sen University, Guangzhou, China; ^3^ Department of Thyroid, Breast and Vascular Surgery, Xijing Hospital, the Air Force Medical University, Xi’an, China; ^4^ Department of Oncology, The Second Affiliated Hospital of Xi’an Jiaotong University, Xi’an, China; ^5^ Department of Neurology, The Second Affiliated Hospital of Xi’an Jiaotong University, Xi’an, China

**Keywords:** hepatocellular cancer, endoplasmic reticulum stress, gene signature, overall survival, immune infiltrate cells

## Abstract

Liver cancer is the sixth most frequently diagnosed primary malignancy and ranks as the third leading cause of cancer-related death worldwide in 2020. ER stress also plays a vital role in the pathogenesis of malignancies. In the current study, we aimed to construct an endoplasmic reticulum stress-related genes (ERGs) signature to predict the overall survival (OS) of patients with HCC. Differentially expressed ERGs (DE-ERGs) were analyzed using The Cancer Genome Atlas (TCGA-LIHC cohort) and International Cancer Genome Consortium (ICGC-LIRI-JP cohort) databases. The prognostic gene signature was identified by the univariate Cox regression and Least Absolute Shrinkage and Selection Operator (LASSO)-penalized Cox proportional hazards regression analysis. The predictive ability of the model was evaluated by utilizing Kaplan–Meier curves and time-dependent receiver operating characteristic (ROC) curves. Gene set variant analysis (GSVA) was performed to explore the underlying biological processes and signaling pathways. CIBERPORT and single-sample Gene Set Enrichment Analysis (ssGSEA) were implemented to estimate the immune status between the different risk groups. A total of 113 DE-ERGs were identified between 50 normal samples and 365 HCC samples in the TCGA-LIHC cohort, and 48 DE-ERGs were associated with OS through the univariate Cox regression. A six DE-ERGs (*PPARGC1A*, *SQSTM1*, *SGK1*, *PON1*, *CDK1*, *and G6PD*) signature was constructed and classified patients into high-risk and low-risk groups. The risk score was an independent prognostic indicator for OS (HR > 1, *p* < 0.001). The function enrichment analysis indicated that cell cycle, RNA degradation, protein localization, and cell division were the main biological processes. The high-risk group had higher immune cell infiltration levels than those of the low-risk group. We predicted the response to targeted therapy in high- and low-risk patients with HCC and found that the high-risk patients were more sensitive to pazopanib. At last, we verified the expression of the six gene patterns in HCC tissues by qRT-PCR and immunohistochemistry. This signature may be a potential tool to provide a choice for prognosis prediction and personal management of patients with HCC.

## Introduction

Liver cancer is the sixth most frequently diagnosed primary malignancy and ranks as the third leading cause of cancer-related death worldwide in 2020 (Sung et al., 2021). Hepatocellular carcinoma (HCC) is the most common form of liver cancer and accounts for 90% of the deaths (Llovet et al., 2021). At present, surgery is still the most effective treatment for HCC. However, due to the occult onset and rapid progression, patients often have lost the best opportunity for surgical treatment at the time of diagnosis. Moreover, the patients with HCC have a poor prognosis because of highly distant metastasis and recurrence rate (Forner et al., 2018), with a 5-year survival rate of only 18.1% (Jemal et al., 2017). Therefore, it is essential to explore the molecular mechanism of HCC development and find new early diagnosis and treatment targets for HCC patients.

Endoplasmic reticulum (ER) is the largest organelle in eukaryotic cells, which functions in protein synthesis and transportation, protein folding, lipid and steroid synthesis, carbohydrate metabolism, and calcium storage (Fagone and Jackowski, 2009). Pathological or physiological stress such as oncogene activation, iron imbalance, oxidative stress, nutritional deficiency, calcium homeostasis disorder, viral infection, exceed protein secretion, and hypoxia can interfere with the normal protein folding process of ER, resulting in the accumulation of unfolded/misfolded proteins in the ER lumen and leading to ER stress (Ma and Hendershot, 2004; He et al., 2020). Importantly, ER stress is mainly coordinated by three sensors, namely PRKR-like ER kinase (PERK), inositol requiring enzyme 1 (IRE1), and activating transcription factor 6 (ATF6), which properly reduce the load of unfolded proteins to reinstate the cell homeostasis (Xu et al., 2021). Moreover, ER stress plays a vital role on the pathogenesis of malignancies (Wadgaonkar and Chen, 2021). Increased protein synthesis caused by ER stress leads to unregulated cell proliferation, which is involved in the occurrence and development of solid tumors (Marciniak et al., 2021). In addition, the PERK inhibitor GSK2656157 can efficiently reduce cancer growth (Atkins et al., 2013). While some preclinical *in vivo* and *in vitro* approaches have shown promising results by targeting ER stress-related molecules such as IRE1α and PERK, its specific mechanism and its relationship with other related pathways are still unclear.

Links between ER stress and tumor immune microenvironment (TIME) are firmly established.

TIME plays an important role in regulating tumor progression. The interaction between cellular and structural components modulates cancer cell invasion and promotes cancer metastasis (Neophytou et al., 2021). Hepatocytes are enriched with ER and are susceptible to ER stress, which contributes to a passive immune response and participates in the development of aggressive and drug-resistance hepatocellular carcinoma. Furthermore, checkpoint-blockade immunotherapies have radically reversed cancer therapy (Wei et al., 2021). Immunotherapy targeting cytotoxic T lymphocyte antigen 4 (CTLA4), programmed cell death-ligand 1 (PD-L1), or programmed cell death 1 (PD-1) have become effective and frequently-used ways in the treatment of various cancers (Herbst et al., 2019; Pires da Silva, 2021; Sacco, 2021). In addition, tumor mutation burden (TMB) refers to the total number of somatic mutations in the tumor cells, and increased TMB may carry neoantigens to stimulate anti-cancer immunity response (Schumacher et al., 2015; Roszik et al., 2016). A previous study reported that TMB predicts survival after immunotherapy across multiple cancer types (Samstein et al., 2019). However, the prediction of TMB for immunotherapy in HCC patients is inaccurate. A challenging problem which arises in this domain is that its suitable population and mechanism in HCC remain unclear.

In this study, we firstly constructed a clustering analysis based on DE-ERGs from the TCGA-LIHC cohort. Moreover, we fortunately built and validated a risk model to predict the outcomes of HCC patients from TCGA and ICGC databases. Moreover, we explored the correlation between the risk signature and TIME and TMB. Importantly, we verified the expression of the six-gene patterns in HCC tissues by qRT-PCR and immunohistochemistry. Furthermore, we analyzed the association between GDSC drug sensitivity and the ERGs-related risk model. These results may prove a new insight for HCC survival prediction and therapy strategies.

## Materials and Methods

### Data Selection

RNA-seq and clinical information of patients with HCC were obtained from The Cancer Genome Atlas (TCGA) data portal (TCGA-LIHC cohort-FPKM) (http://portal.gdc.cancer.gov/) and International Cancer Genome Consortium (ICGC-LIRI-JP) database (http://daco.icgc.org). The comprehensive gene list of ERGs was extracted from Genecard (https://www.genecards.org/), and genes with a relevance score ≥7 were chosen (Zhang et al., 2021). TCGA-LIHC mutation data (TCGA. LIHC. varscan somatic) was also downloaded from the TCGA database.

### Differentially Expressed Endoplasmic Reticulum Stress-Related Genes Analysis

To choose ERGs that contribute to the development and progression of HCC, differentially expressed genes (DEGs) between tumor tissue and normal tissues were analyzed using the “edgeR” package. The DEGs with an adjusted *p*-value < 0.05 and |log2 (fold change) | > 1 were considered as screened criterion. Differentially expressed ER stress-related genes (DE-ERGs) were identified by the intersection between the ERGs list (mentioned above) and the DEGs list through the online tool Jvenn (http://jvenn.toulouse.inra.fr/). The “clusterprofiler” package in R software was utilized for Gene Ontology (GO) and Kyoto Encyclopedia of Genes and Genomes (KEGG) enrichment analysis to identify the biological function of DE-ERGs in HCC (Kanehisa et al., 2012; Gene Ontology Consortium, 2015). Adjusted *p* value < 0.05 was set as significant screen criteria.

### Identification of Molecular Subgroups of Hepatocellular Carcinoma

We used the STRING database (https://string-db.org) to explore the degree of interactions among the DE-ERGs, and the interaction combined score >0.4 was defined as a significant edge score (Szklarczyk et al., 2015). Then, cytoHubba of Cytoscape (Version 3.8.2) was used to visualize the top 20 hub genes in the PPI network (Shannon et al., 2003). In addition, we performed the Non-negative Matrix Factorization (NMF) method to identify subgroups of HCC samples based on 113 DE-ERG transcription profiles using the “NMF” R package. The samples were iterated thirty times through NMF, extracting the biological correlation coefficient and predicting the internal characteristic structure in the gene expression matrix (Gaujoux and Seoighe, 2010). Using the cophenetic coefficient, contour, and sample size algorithm, the HCC samples were classified into k clusters with k = 2–10, and the samples were divided into two categories.

### Development and Reliability Evaluation of Prognosis-Related Signature

The prognosis-related DE-ERGs were identified and a six ERGs risk score signature was developed based on the training set, and its predictive performance was validated in the test dataset. The univariate cox regression analysis was used to identify genes related to survival with adjusted *p*-values <0.05*.* Then the significant genes were selected for the least absolute shrinkage and selection operator (LASSO) cox regression, which was performed by using the “glmnet” R package (Friedman et al., 2010; Wang et al., 2019). The regression coefficients were derived from the LASSO cox analysis and the risk score= (β 1 * EXP _gene1_) + (β2 * EXP _gene2_) +…+ (β n* EXP _gene n_) (Liang et al., 2020). The patients were classified into high-risk and low-risk groups based on the median value of the risk score. The Kaplan–Meier survival curve, and the area under the curve (AUC) of the time-dependent receiver operating characteristics (ROC) curve were applied to estimate the predictive ability of the prognostic model. The principle component analysis (PCA) was performed to explore whether two risk groups were distributed in discrete directions. The independent predictive efficiency of the prognostic signature was evaluated by univariate and multivariate cox analyses. The Mann–Whitney test was applied to evaluate the association of risk signature with different clinicopathologic features using GraphPad Prism 8. A bilateral *p* value < 0.05 was of statistical significance. The hazard ratio (HR) and 95% confidence intervals (CI) were calculated. The patients with survival information from ICGC-LIRI-JP were used for external validation. The same methods were performed to assess risk scores for each case.

### Gene Set Variant Analysis and Mutation Analysis

The “GSVA” and “clusterProfiler” package in R was utilized to evaluate the Gene Oncology biological processes and KEGG pathways of this signature. The R package “limma” was applied to screen the significant terms with an adjusted *p* value <0.05. We identified the different biological pathways enriched in the different risk groups. We explored the mutation status between the two risk groups. The “maftools” R package was applied to visualize the TCGA-LIHC mutation data and drew the waterfall plots of high- and low-risk groups (Mayakonda et al., 2018).

### Estimation of TME, Tumor-Infiltrating Immune Cell Types and Immune Checkpoint in Hepatocellular Carcinoma Patients

To further assess the correlation between the risk score and TME, ESTIMATE was performed to calculate the stromal score and immune scores that presented immune cell infiltration in the tumor (Yoshihara et al., 2013). We used the “GSVA” R package to perform ssGSEA analysis, and obtained the infiltrating scores of 16 immune cells and the immunoactivity of 13 immune-related pathways in the HCC patients (Hänzelmann et al., 2013). We utilized the “heatmap” R package to show the expression feature of the known immune checkpoints on HCC patients in different risk groups.

### Prediction of Drug Sensitivity in Risk Models

The correlation between drug sensitivity and the mRNA expression of six DE ERGs was investigated through the web server GSCALite (http://bioinfo.life.hust.edu.cn/GSCA). Moreover, the response of targeted therapy in HCC patients was determined based on the public database GDSC (Genomics Drug sensitivity in cancer) (Yang et al., 2013). The half-maximal inhibitory concentration (IC50) was evaluated to represent the drug response. In addition, the package “pRRophetic” was applied to estimate the potential target drug response between high-risk and low-risk groups.

### Tissue Samples and Real-Time PCR and Immunohistochemical Staining

We collected 33 pairs of HCC and para-cancer tissues from HCC patients who underwent hepatectomy in Sun Yat-Sen Memorial hospital between December 2021 and March 2022 and stored them in 80 refrigerators. The clinical information of these patients was also collected (Supplementary Table S6). Informed consent was obtained from each patient, and the study was approved by the Ethics Committee of Sun Yat-Sen Memorial. We extracted the RNA from the tissues with Trizol (Takara, China), and performed reverse transcription using Prime Script RTase (Takara, China), according to the manufacturer’s protocol. According to the manufacturer’s instructions, real-time PCR was used to measure mRNA expression levels using SYBR green (Takara, China). A list of the primers used for real-time PCR is provided in Supplementary Table S7. Immunohistochemical (IHC) staining was performed as described previously (Zhang et al., 2020) using the following antibodies: Anti-PPARGC1A, Anti-PON1, Anti- SGK1, Anti- SQSTM1, Anti- G6PD, and Anti-CDK1. All antibodies used in the study are shown in Supplementary Table S8.

### Statistics Analysis

Differences between the high- and low-risk groups were tested using the Mann–Whitney test for non-normally distributed variables and the unpaired *t*-test for normally distributed variables. The correlation between gene expression and risk score was tested using the Pearson correlations. The statistical analysis tools-R software (version 4.0.3, R Foundation for Statistical Computing, Vienna, Austria) and GraphPad prism v8.00 (GraphPad Software Inc.) were used in this study. Venn diagram was drawn using jvenn (Bardou et al., 2014), and the results of RT-qPCR were conducted statistical analysis using paired *t*-test. All statistical results with a *p*-value of <0.05 were considered significant.

## Results

### Data Source

The TCGA-LIHC cohort containing 424 samples (374 patients with HCC and 50 normal samples) was used to analyze differentially expressed genes. We obtained 365 samples with detailed clinical characteristics (age, sex, survival time, survival status, pathological grade, and TNM stage) to further estimate the independence of the predictive model. The 231 Japanese patients were from the ICGC-LIRI-JP dataset. The basic description of patients’ clinical characteristics used in this study is presented in Supplementary Table S1. There are 786 ER stress-related genes with a relevance score of not less than seven listed in Supplementary Table S2.

### Screening of Differentially Expressed Endoplasmic Reticulum Stress-Related Genes in Hepatocellular Carcinoma Patients

**FIGURE 1:**
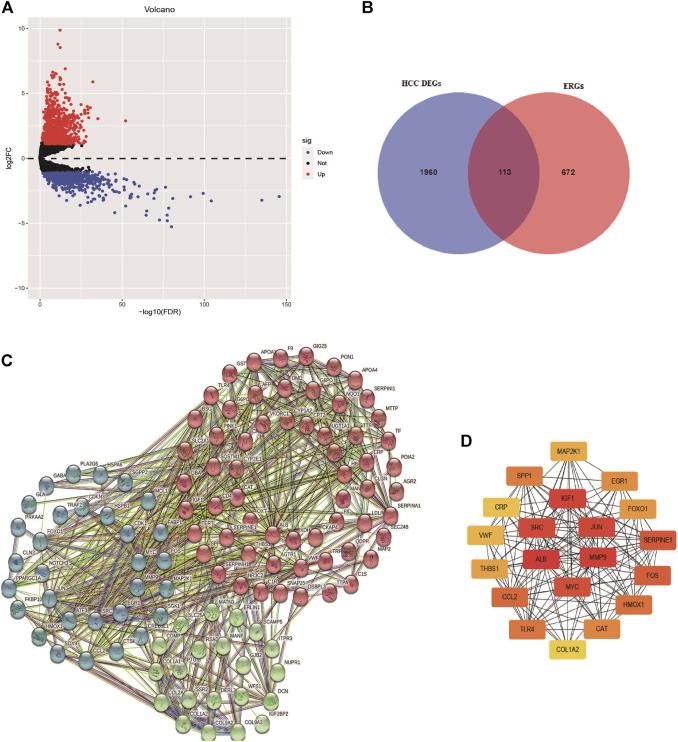
Identification of differentially expressed endoplasmic reticulum stress-related genes. **(A)** Volcano plot of differentially expressed genes in HCC based on data from TCGA-LIHC cohort. **(B)** Venn diagram for the intersections between HCC differentially expressed genes and endoplasmic reticulum stress-related genes. The interaction network among these genes is exhibited in **(C)**, and the top 20 hub genes are presented in **(D)**.

Based on the screened criteria, 2,073 DEGs were selected: 1,298 were upregulated and 775 were downregulated (Figure 1A). From this list of ERGs, 113 DE-ERGs were extracted (Figure 1B). The results of GO analysis for these DE-ERGs were presented in Supplementary Figure S1A, in which the most enriched terms were “cellular response to stress,” “endoplasmic reticulum lumen,” and “extracellular matrix structural constituent,”, respectively. The most important enriched cancer-related signaling pathways by these genes were: the “PI3K-AKT signaling pathway, the “HIF-1 signaling pathway”, and the “TNF signaling pathway” (Supplementary Figure S1B). The interaction network among these genes is exhibited in Figure 1C. Cancer-associated proteins, such as *MYC*, *IGF-1,* and *MMP9* were identified as the main hubs in the resulting networks, and it had been previously reported that they were related to tumorigenesis and metastasis (Figure 1D).

### Identification of Hepatocellular Carcinoma Subtypes Based on Differentially Expressed Endoplasmic Reticulum Stress-Related Gen

**FIGURE 2:**
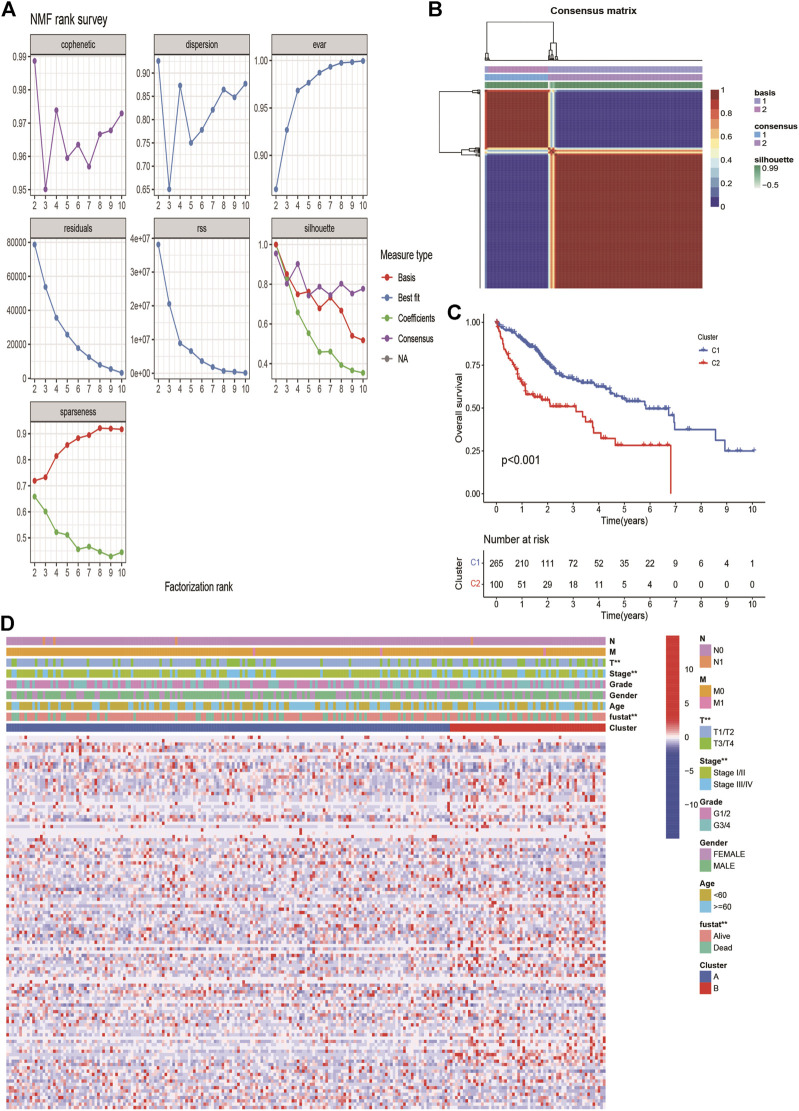
Non-negative Matrix Factorization of HCC molecular subgroups based on DE ERGs. **(A)** The curves of cophenetic correlation coefficient, RSS, and dispersion et al. were used to reflect the stability of the cluster obtained from NMF. **(B)** The heatmap corresponding to the consensus matrix for k = 2 obtained by applying NMF. **(C)**. K-M survival curves showed the differences of overall survival rate among the 2 clusters. **(D)** The heatmap of 113 endoplasmic reticulum stress-related genes in 2 clusters.

The expression profiles of 113 DE-ERGs were used for NMF analysis of HCC. According to the cophenetic coefficient (Figure 2A), the optimum cluster was obtained when the k-value was 2. The 365 HCC patients were divided into two clusters: cluster 1 (*n* = 265) and cluster 2 (*n* = 100; Figure 2B). A survival analysis revealed that cluster two had a poorer prognosis than that of cluster one (*p* < 0.001, Figure 2C). The expression landscapes of 113 DE-ERGs in cluster 1 and 2 with different clinical features were presented in the heatmap (Figure 2D), and there were significant differences between the two clusters and tumor size, tumor stage, and patients’ survival status.

### Construction and Verification of Endoplasmic Reticulum Stress-Related Gene Signature

**FIGURE 3:**
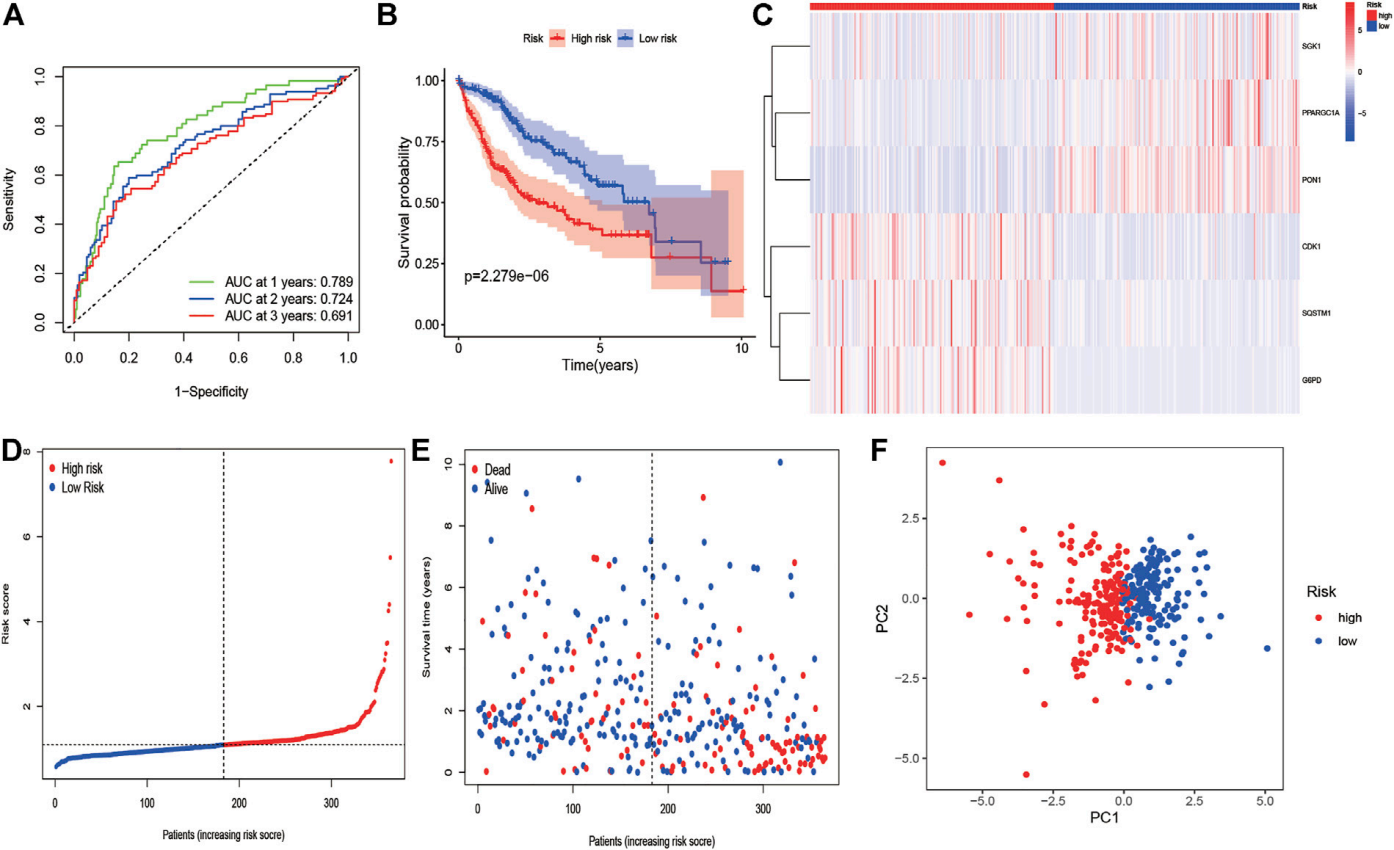
KM survival analysis, risk score assessment by the ERG-related gene signature and time-dependent ROC curves in the training cohort. **(A)** ROC curve for overall survival of the training set. The AUC was assessed at 1–3 years. **(B)** KM survival analysis of high-and low-risk samples. **(C)** Six ERGs expression patterns for patients in high- and low-risk groups by the 6-GRG signature. **(D, E)** Relationship between the risk score rank/survival status and risk score rank/survival time (days). **(F)** PCA analysis for HCC patients.

**FIGURE 4:**
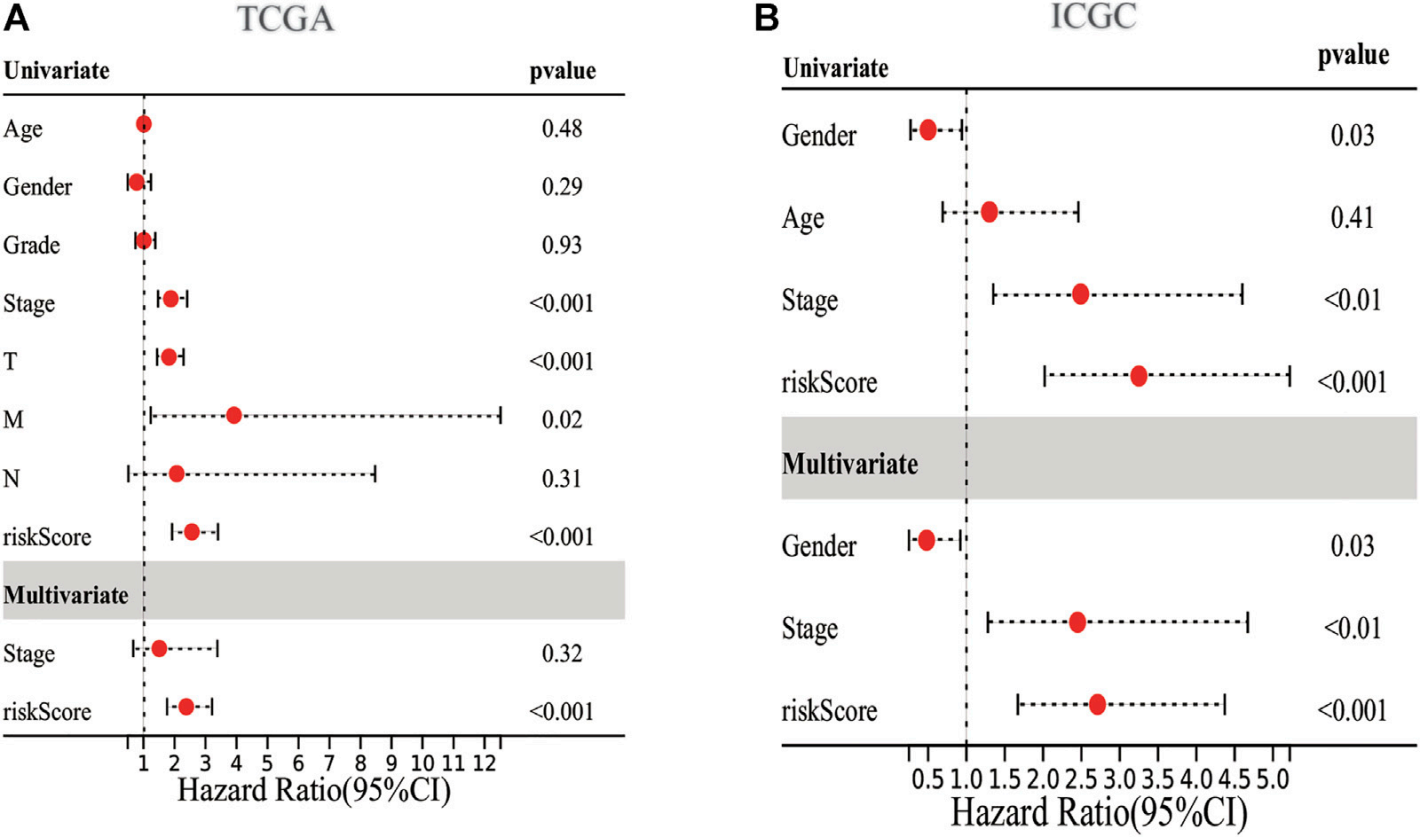
Forrest plot of the univariate and multivariate association of the prognostic model and clinicopathological characteristics with overall survival. **(A,B)** Univariate and multivariate analysis with Cox proportional hazard model in TCGA-LIHC cohort and ICGC-LIRI-JP cohort, respectively.

We employed the univariate Cox analysis to explore survival-related DE ERGs in the training group; 48 DE ERGs related to OS were screened (Supplementary Figure S2A). These 48 prognostic genes were checked in the 1000-times-repeated LASSO-Cox regression model, and then those genes with a frequency of over 1,000 times were constructed to an EGRs signature. The cross-validation for tuning parameter selection in the LASSO model obtained six prognostic genes using minimum λ (Supplementary Figures S2B,C). Six genes (*PPARGC1A*, *SQSTM1*, *SGK1*, *PON1*, *CDK1*, and *G6PD*) were included, as presented in Supplementary Table S3. Three genes (*SQSTM1*, *G6PD*, and *CDK1*) were classified into risky group with HR >1 related to poorer prognosis and presented higher expression levels in tumor tissues than in normal tissues. While others (*PPARGC1A*, *PON1*, and *SGK1*) were the protective type with HR <1 related to a better prognosis, and preferentially lower expressed in malignant tissues than normal tissues, but *SGK1* had no significant result in survival (Supplementary Figures S3, S4). We built a prognostic model based on the results of LASSO regression to explore the relationship between the six ERGs signature and survival. The risk score was calculated using the following formula: risk score= (−0.0060 × EXP_PPARGC1A_) + (−0.0013 × EXP _PON1_) + (−0.0010 × EXP_SGK1_) + (0.0013 × EXP _SQSTM1_) + (0.0089 × EXP_G6PD_) + (0.0181 × EXP_CDK1_). The patients in the training cohort were assigned to the high-risk group (*n* = 182) or low-risk group (*n* = 183) according to the median value of the risk score. The clinical characteristics of HCC patients in the different subgroups were displayed in Supplementary Table S4. The AUCs of the risk score were 0.789, 0.724, and 0.691 for the 1–3-years survival times, respectively (Figure 3A). The K-M curve indicated that the patients in the high-risk group exhibited poorer OS than that of the low-risk group (*p* = 2.27e-06; Figure 3B). The heatmap revealed expression patterns of six ERGs between two different risk groups (Figure 3C). We ranked the risk score of patients in the training cohort and analyzed their distribution in Figure 3D. The survival status of HCC patients in the training set was marked on the dot plot (Figure 3E). With increasing the risk score, the number of dead patients increased. We confirmed that patients in the two risk groups were distributed in discrete directions (Figure 3F). To assess the robustness of the six ERGs signature, we verified their performance using the validation cohort from ICGC. Similarly, the 1–3-year AUCs were 0.735, 0.738, and 0.713, respectively (Supplementary Figure S5A). The patients in the high-risk group had a poorer prognosis than that of the low-risk group (*p* = 0.01485; Supplementary Figure S5B). The distribution of PI, survival status, and expression of these six ERGs for HCC patients in the test set were displayed in Supplementary Figures S5C–E. Furthermore, univariate and multivariate Cox regression models were used to analyze the relationship between OS, clinical-pathological variables, and the risk scores in the training and validation cohort (Figure 4; Supplementary Table S5). This risk signature could act as an independent prognostic factor for OS through the multivariate analysis. The differences between the different risk groups and clinical features were evaluated in the TCGA-LIHC cohort and ICGC-LIRI-JP cohort (Supplementary Figure S6). The six ERGs signature was significantly higher in advanced grade, AJCC stage, and T stage cases in the TCGA-LIHC cohort. However, no difference was observed between age, gender, N stage, and M stage. Survival analysis of subgroups revealed that the high-risk group had a shorter survival time than the low-risk group, whatever age, gender, clinical stage, pathological grade, or TNM stage (Supplementary Figure S7).

### Gene Set Variant Analysis and Genomic Mutations in the High-Risk and Low-Risk Group

**FIGURE 5:**
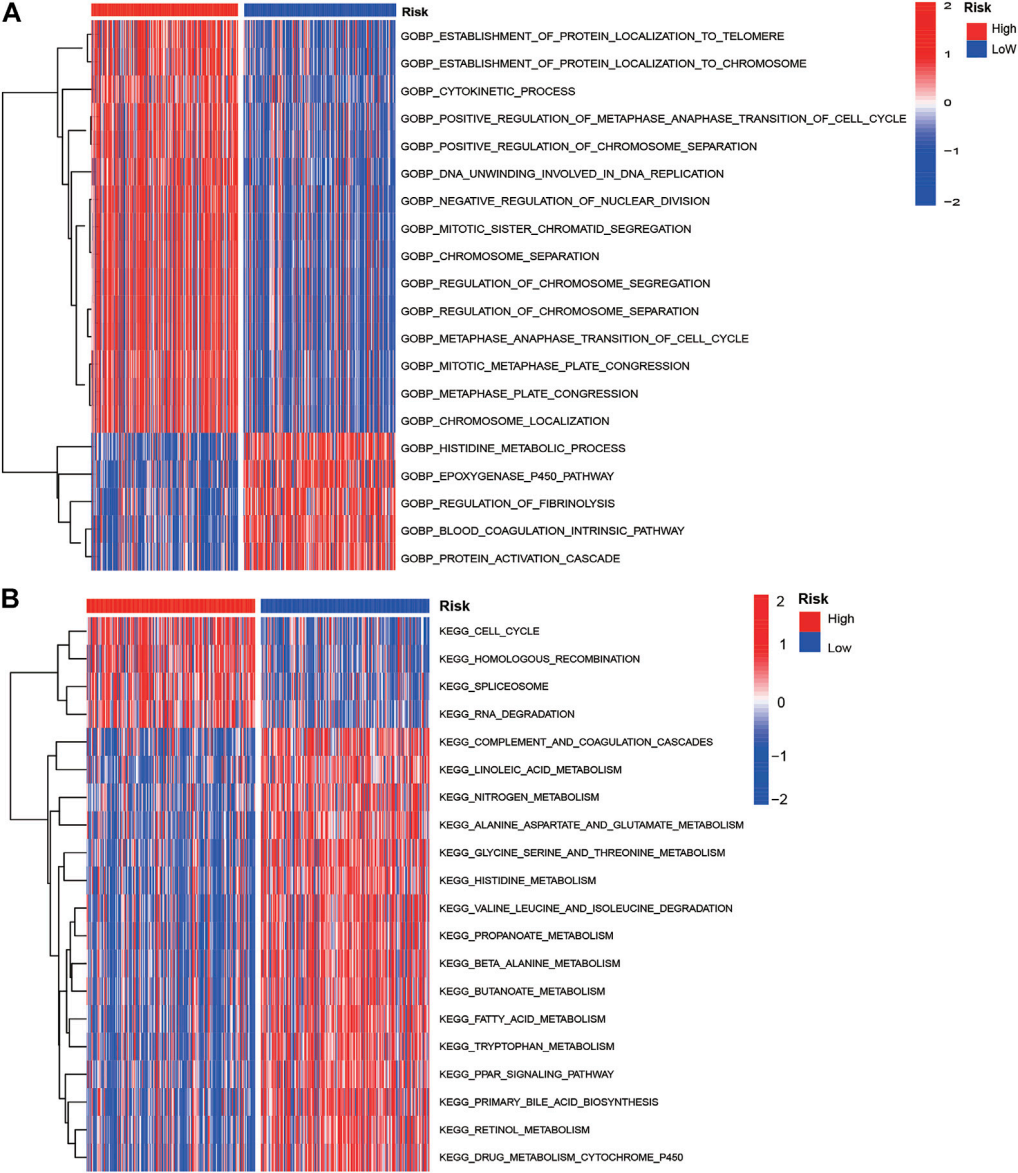
GO biological process terms **(A)** and KEGG pathways **(B)** enrichment of low- and high-risk groups by GSVA analyses in the training cohort.

The GSVA analysis showed most KEGG pathways and GO biological process terms enriched in the high-risk group were associated with cell cycle, RNA degradation, protein localization, and cell division. The oncogenesis-associated signaling pathways such as Wnt beta-catenin signaling and PI3K/AKT/mTOR signaling were highly enriched in the high-risk group, while oxidative phosphorylation and fatty acid metabolism were enriched in the low-risk group (Figures 5A,B). The top 20 most frequently mutated genes in 298 patients with HCC were presented in Supplementary Figures S8A,B. As shown, we can see the mutation genes and mutation frequencies were different between two risk groups. The high-risk group exhibited higher mutation frequencies than those of the low-risk group (90 vs. 81%). The high-risk group had a significantly higher mutation frequency of TP53 than that of the low-risk group (41 vs. 14%). Interestingly, we found that TMB was higher in high-risk group (*p* < 0.0001) but not associated with OS (*p* = 0.108; Supplementary Figures S8C,D).

### Tumor-Infiltrating Immune Cell Types and Immune Checkpoint Feature of Risk Signature

**FIGURE 6:**
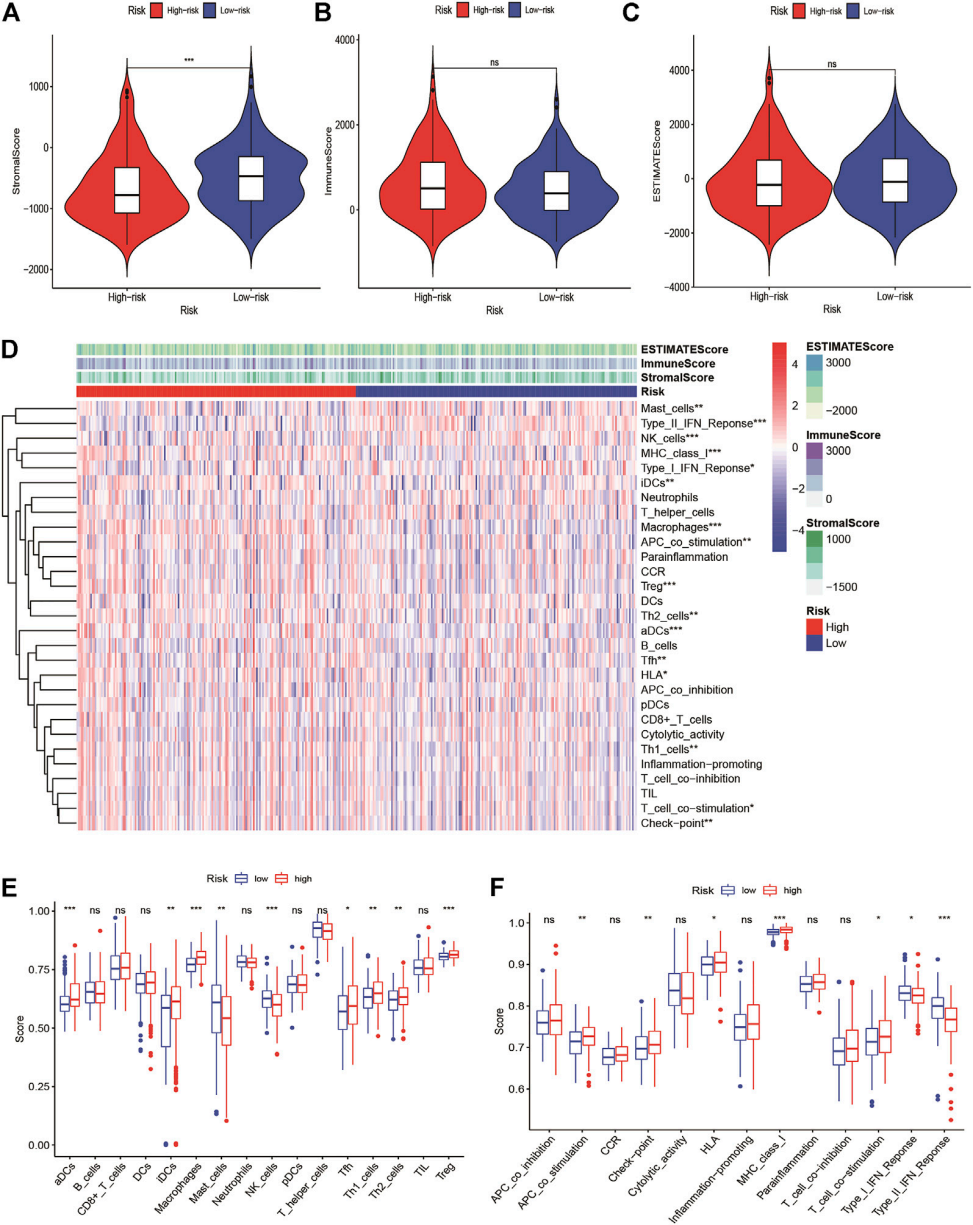
Tumor microenvironment in high-risk and low-risk groups. Comparison of the **(A)** stromal score, **(B)** immune score, and **(C)** ESTIMATE score between high-risk and low-risk groups **(D)** The immune landscapes of high-risk and low-risk groups. **(E)** Comparison of immune cell infiltration between high-risk and low-risk groups. **(F)** Comparison of the involved immune related function between high-risk and low-risk groups. *Adjusted *p* < 0.05, ** adjusted *p* < 0.01, *** adjusted *p* < 0.001, **** adjusted *p* < 0.0001.

We found that the immune score was higher in the high-risk group but of no significance, and the stromal score was higher in the low-risk group (Figures 6A–C). We quantified the enrichment scores of diverse tumor-infiltrating immune cell types and explored related functions or pathways with ssGSEA. The heatmap of 16 types of immune cells and 13 immune-related functions in each HCC patient were displayed in Figure 6D. We found that contents of the antigen-presented process, including aDCs, iDCs, Macrophages, Tfh, Th1 cells, Th2 cells, and Treg cells enriched in the high-risk group. Moreover, these related pathways APC co-stimulation, HLA, checkpoint, MHC class I, T cell co-stimulation had a higher score in the high-risk group, while the score of NK cells, type I IFN response and type II IFN response were higher in the low-risk group (Figures 6E,F). The expression of immune checkpoints between the high- and low-risk groups showed that most immune checkpoints were high-expression levels in the high-risk group in the TCGA-LIHC cohort and ICGC cohorts (Supplementary Figures S9A,B). At present, the immunotherapy targeting PD-1, PD-L1, and CTLA4 has shown remarkable efficacy on tumor immune activation. Therefore, we investigated the correlation between their expression levels and the risk score. We found that the expression levels of PD-1 and CTLA4 were higher in the high-risk group than in the low-risk group, and their expression levels were positively correlated with the signature. However, the expression level of PD-L1 has no statistical significance (Supplementary Figures S10A–L).

### Correlation Between Genomic Drug Sensitivity in Cancer Drug Sensitivity and the Risk Model

**FIGURE 7:**
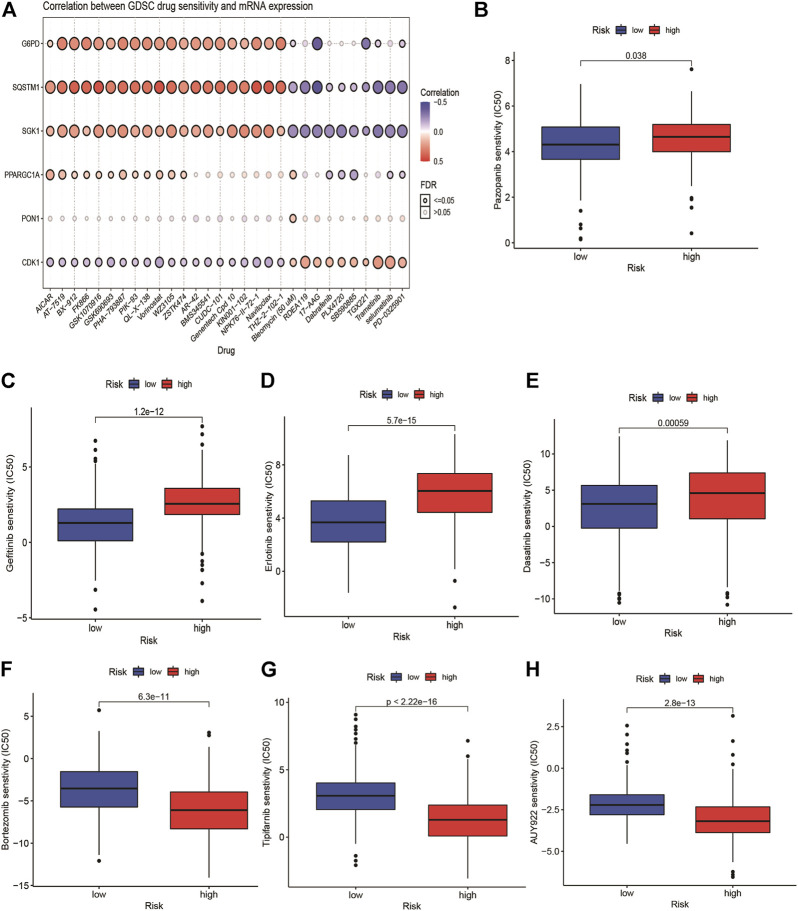
Relationship of ERG-related risk model with target therapy response. **(A)** the correlation between GDSC drug sensitivity and the risk score. Estimated IC50 indicating the efficiency of chemotherapy to ERGs in low- and high-risk patients of pazopanib **(B)**, dasatinib **(C)**, erlotinib **(D)**, bortezomib **(E)**, Tipifarnib **(F)**, gefitinib **(G)** and AUY929 **(H)**.

We explored the correlation between GDSC drug sensitivity and the risk score. The results from GSCA suggested that there was an apparent correlation between the expression of G6PD, SQSTM1, and SGK1 and multiple drug sensitivity (Figure 7A). Furthermore, we observed that seven targeted therapy drugs including pazopanib, dasatinib, erlotinib, bortezomib, tipifarnib, gefitinib, and AUY929 (HSP90 inhibitor) witnessed significant differences in estimated IC50 between high- and low-risk groups (Figures 7B–H). The patients with high risk presented higher sensitivity in pazopanib, gefitinib, erlotinib, and dasatinib than patients with low-risk.

### Validation of the Expression Levels of Endoplasmic Reticulum Stress-Related Genes in Hepatocellular Carcinoma

**FIGURE 8:**
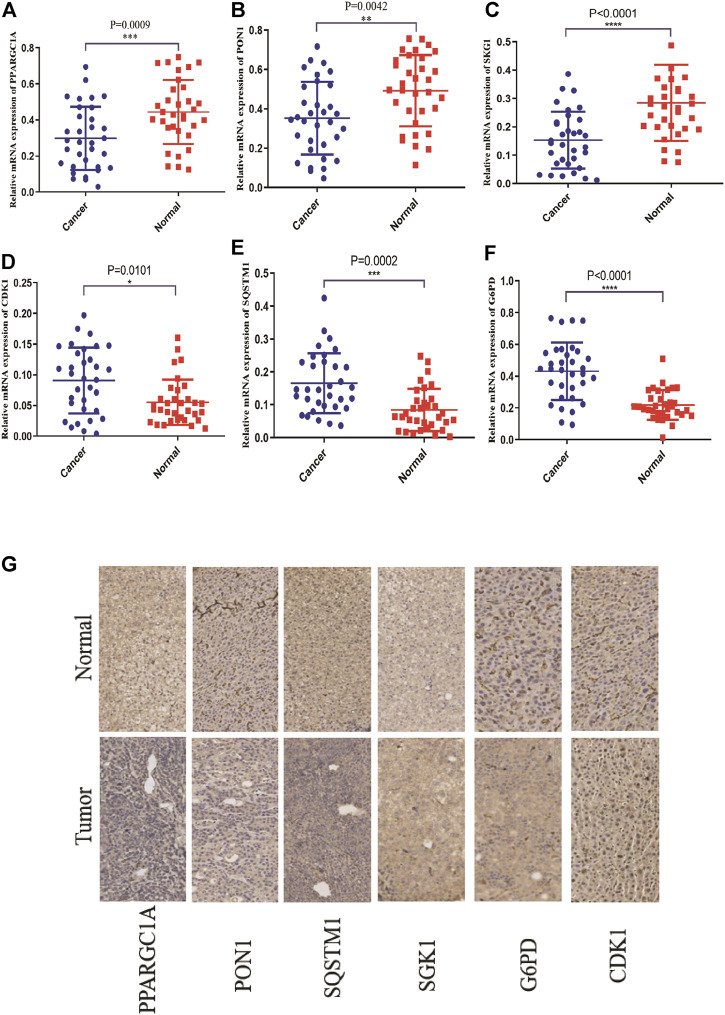
The mRNA and protein expression levels of six ER stress-related genes in the normal tissues and cancer tissues of the HCC patients through qRT-PCR and IHC. **(A–F)** The mRNA expression levels of six ER stress-related genes. **(G)** The protein expression of six ER stress-related genes.

To illuminate the biological significance of ERGs in HCC, we used immunohistochemistry and qRT-PCR to detect the expression of ERGs in 33 HCC samples. As presented in Figure 8, the expression levels of PPARGC1A, PON1, and SGK1 were down-regulated in HCC tissues compared to paired normal tissues, while the expression levels of SQSTM1, G6PD, and CDK1 were increased.

## Discussion

At present, ERGs have an important impact on cancer progression, including cell proliferation, invasion, cell death, and metastasis. However, for the majority of ERGs, the biological mechanisms are still unclear in HCC. The identification of ERG’s predictive signature is vital to understand the characterization of endoplasmic reticulum stress in HCC. In this study, we aimed to explore the expression profiles of ERGs in HCC and normal tissues and estimate their roles in tumorigenesis and tumor immunity.

Based on the expression profiles of the training set, we constructed a risk score using univariate and LASSO Cox regression analyses, which showed that the prognostic signature is an effective way to independently generate prognosis of HCC patients. Moreover, we found the high-risk group had a poorer prognosis. This signature was composed of six DE-ERGs (*PPARGC1A*, *SQSTM1*, *SGK1*, *PON1*, *CDK1*, and *G6PD*) with prognostic capability. Three genes (*SQSTM1*, *G6PD*, and *CDK1*) were upregulated in the tumor tissues compared to the HCC normal tissues in the TCGA-LIHC dataset. Three genes (*PPARGC1A*, *PON1*, and *SGK1*) were preferentially lower expressed in malignant tissues than in normal tissues. In addition, our experimental results from qRT-PCR and IHC confirmed this trend. *PPARGC1A,* also named *PGC-1* (alpha), is a transcriptional coactivator that regulates the genes involved in energy metabolism. *PGC-1* (alpha) presented downregulation in prostate cancer and was associated with the development of metastasis (Valcarcel-Jimenez et al., 2019). A previous study had demonstrated that *PGC-1* (alpha) incurred a progressive loss in tumor-infiltrating T cells, and this was induced by chronic AKT signaling in tumor-infiltrating T cells (Scharping et al., 2016). Paraoxonase-1 (*PON1*), an esterase with a broad range of substrate specificity, belongs to a member of the family of paraoxonases. *PON1* activity has been reported in atherosclerosis and cardiovascular disease (Mackness and Mackness, 2004). *PON1* was found to downregulate in gastroesophageal cancers and associated with lymph node metastasis (Krzystek-Korpacka et al., 2008). SGK1 is involved in the development of almost all tumors and may function as a potential biomarker for cancer diagnosis and prognosis. *SGK1* plays multiple roles in the tumor, such as tumorigenesis, cancer cell proliferation, apoptosis, invasive, and migration (Liu et al., 2018). Moreover, an increasing number of studies have suggested that *SGK1* can regulate the functions of immune cells including T helper cells, and regulatory T cells in the tumor microenvironment (Sang et al., 2020). *SQSTM1* (better known as p62), is an autophagy receptor, and its activity mediates multiple biological functions including autophagy, cell growth, and cell death (Gong et al., 2021). Recent studies have demonstrated that *SQSTM1* promotes cell growth and induces autophagy in thyroid cancer by modulating AKT/mTOR signaling pathway (Yu et al., 2021). *G6PD* is a key enzyme in glucose metabolism and plays an important role in the modulation of proinflammatory responses and oxidative stress in macrophages (Ham et al., 2013). The upregulation of *G6PD* in gastric cancer activates NF-κB signaling to promote cancer cell metastasis (Chen et al., 2021). *CDK1* belongs to cyclin-dependent kinase family and participates in regulating the G2/M phase transition during the cell cycle (Malumbres, 2014). *CDK1* was overexpressed in colorectal cancer and liver cancer and can promote cell proliferation and induce apoptosis (Tong et al., 2021). These previous studies indicated that the six ERGs played crucial roles in cancer progression, which provided some basic support for our research.

Based on the GSVA analysis, the high-risk group was apparently positively related to cell cycle, RNA degradation, and protein localization. Notably, tumor-related signaling pathways such as PI3K, MYC, mTOR, and Wnt were significantly enriched in high-risk group, and continuous activation of these pathways have been demonstrated to be linked with HCC (Tian et al., 2021; Xia et al., 2021; Yi et al., 2021). Endoplasmic reticulum stress-related signaling pathway such as unfolded protein response was significantly enriched in the high-risk group, which further validated that endoplasmic reticulum stress has a close connection with tumor development. Immune checkpoints play a critical role on suppressing the immune system’s ability to kill tumor (Dyck and Mills, 2017). In recent years, immune checkpoint therapy, which targets regular pathways in T cells to take part in immune escape response of cancer, has been a new direction in the field of anticancer after traditional therapeutic methods (Jiao et al., 2020). The result of ssGSEA analysis suggested that the endoplasmic reticulum stress is positively correlated with the immune signaling pathways in HCC patients. The high-risk group has a higher infiltration level of DCs, iDCs, macrophages, and Th2 cells, as well as higher expression levels of immune checkpoints. The result now evidence of the relationship between endoplasmic reticulum stress and immunity, which emphasized the key role of immunotherapy for HCC patients with a high-risk score. In addition, the expression of PD1, and CTLA4 are significantly higher in the high risk-group than in the low-risk group, and are positively correlated with the risk score. Moreover, we found that high-risk group has higher TMB levels. In a word, combined with previous studies, we speculated that high-risk groups tend to benefit from immunotherapy. The mutation frequency of TP53, CTNNB1, TTN, and MUC16 are more than 10% in different risk groups. TP53 is involved in cell cycle, and its mutation may promote tumor procession. We guessed that a higher TMB in the high-risk group induced higher immune cells infiltration and had poorer survival rate, which is in line with the previous study (Schumacher et al., 2015). Overall, this suggests that the prognostic signature can predict the expression level of immune checkpoints. With the progress of HCC, common targeted therapy has become extremely limited. New targeted therapeutic drugs need to be applied to alleviate the advanced HCC patients and improve survival rate. Therefore, we attempted to predict the response to targeted therapy in high- and low-risk patients and found that high-risk patients with HCC were more sensitive to pazopanib (VEGFR inhibitor), dasatinib (Src/Bcr-Abl inhibitor), erlotinib, and gefitinib (EGFR inhibitor) than low-risk patients were. These results suggested patients with high risk can receive greater clinical benefits from targeted therapy.

Nevertheless, the limitations of this study should be addressed. First, this is a bioinformatics analysis based on public cancer databases; hence, the prognostic robustness and clinical utility of the ERGs signature need to be further verified in larger prospective trials. Second, although we verified the expression of six gene patterns in HCC tissue, the more in-depth mechanisms of ERGs and liver cancer should be explored in laboratory settings.

## Conclusion

We integrated the six-ERGs into a panel and established a novel multigene signature for predicting the prognosis in HCC, and further investigated the biological mechanism, TME, and genomic mutation of this prognostic model. This signature may be a potential tool to provide a choice for prognosis prediction and personalized management of HCC.

## Data Availability

The original contributions presented in the study are included in the article/Supplementary Material, further inquiries can be directed to the corresponding authors.
