# Supplementary material for: Identification of an Endoplasmic Reticulum Stress-Related Gene Signature to Evaluate the Immune Status and Predict the Prognosis of Hepatocellular Carcinoma
Source: Front Genet. 2022 May 27;13:850200. doi: 10.3389/fgene.2022.850200 (PMC9197218; doi:10.3389/fgene.2022.850200)
Supplement: Supplementary file 1 [file DataSheet2.docx]

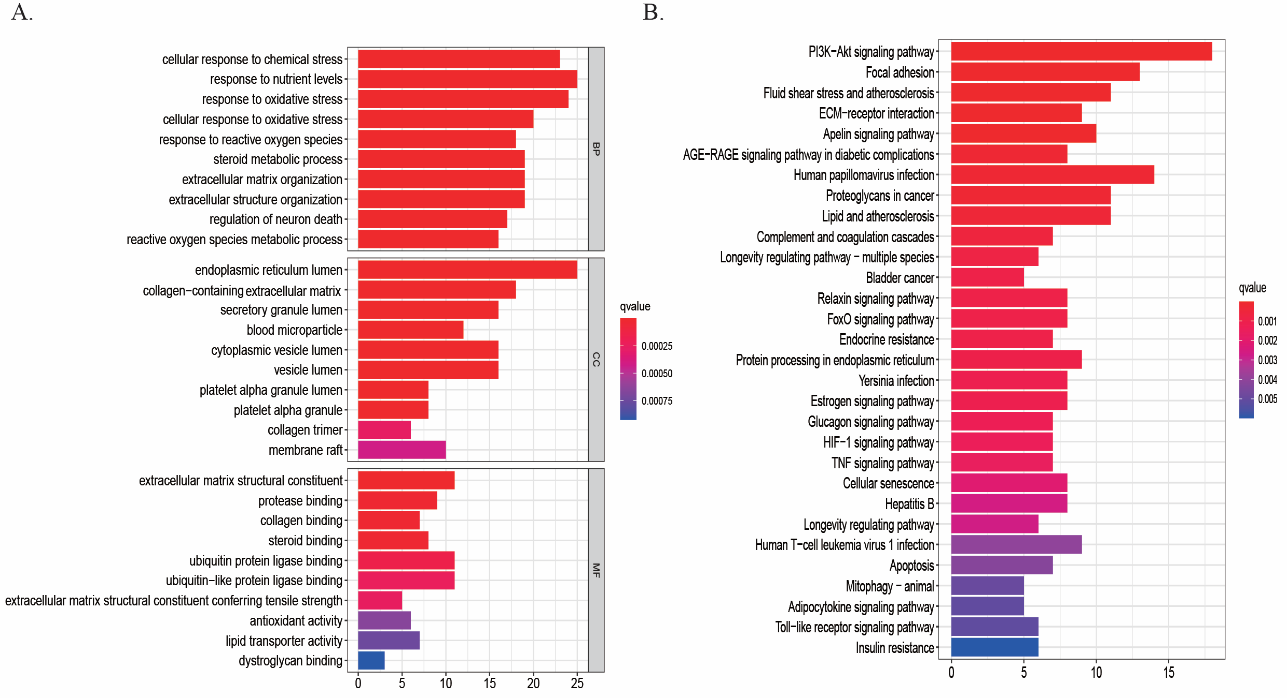


**Figure S1. Functional enrichment analyses of differentially expressed ER stress-related genes. (A) Gene ontology analysis. (B) The top 30 most significant Kyoto**

**Encyclopedia of Genes and Genomes pathways.**


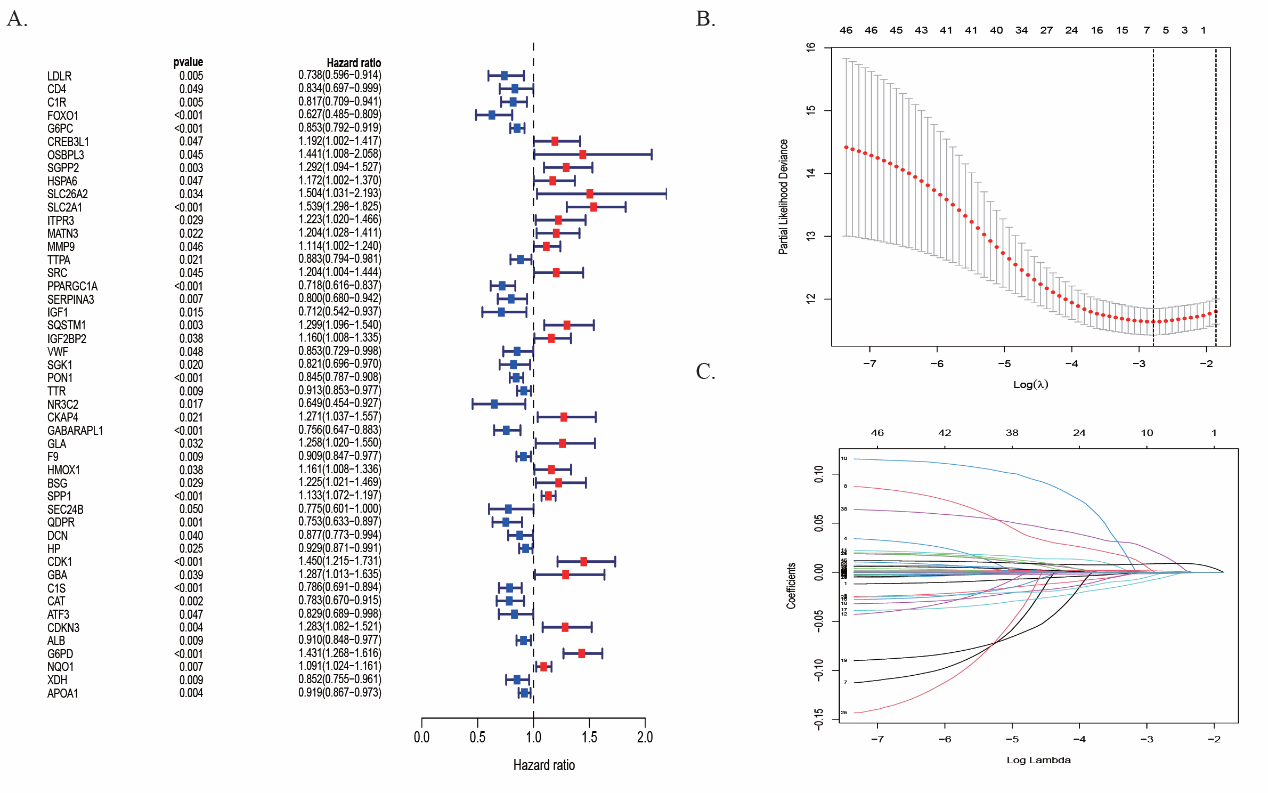


**Figure S2. Construction of a prognostic ERG-related model in TCGA-LIHC.** (A) Forest plot illustrating 48 DE ERGs identified through the univariate Cox analysis. (B). 10-Fold cross-validation analysis of the 6 selected ERGs in the LASSO model. (C). X-tile for the coefficients of 6 ERGs of HCC.


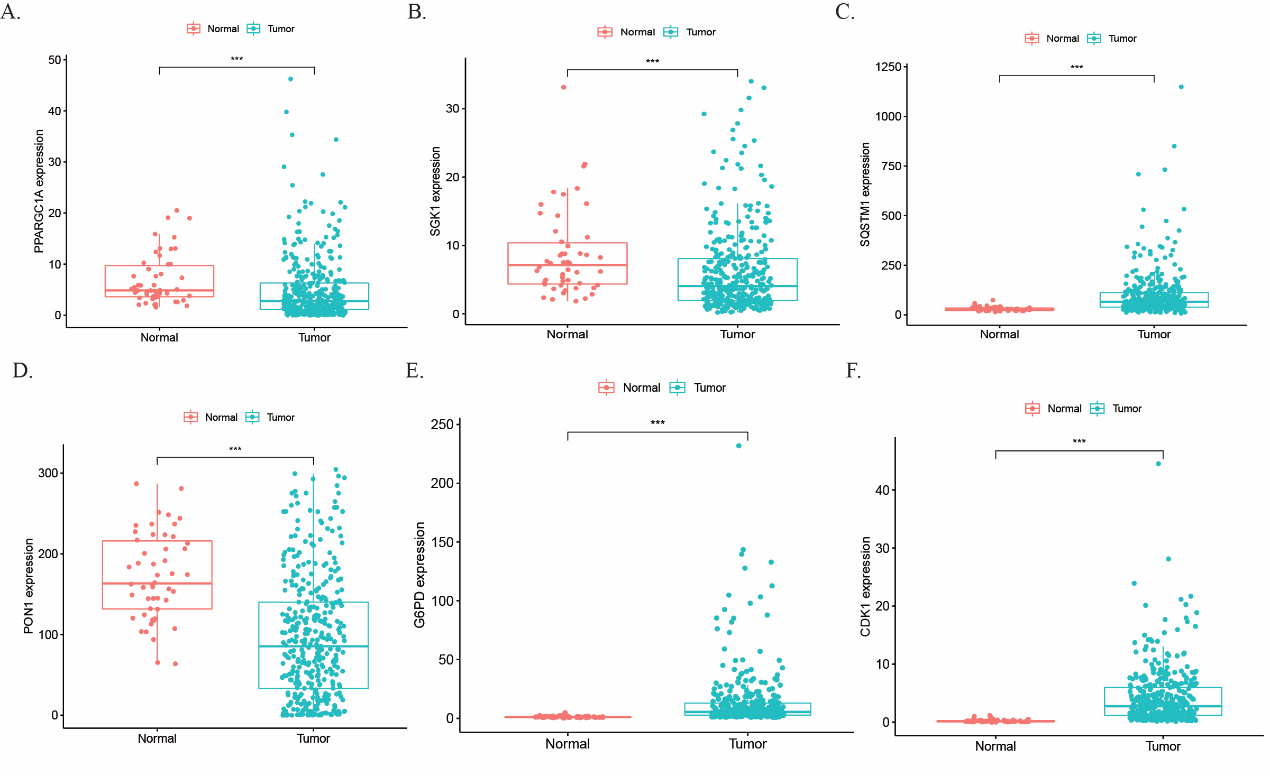


**Figure S3. The expression levels of 6 ER stress-related genes in the high- and low-risk subgroups in the TCGA-LIHC COHORT-LIHC cohort.**


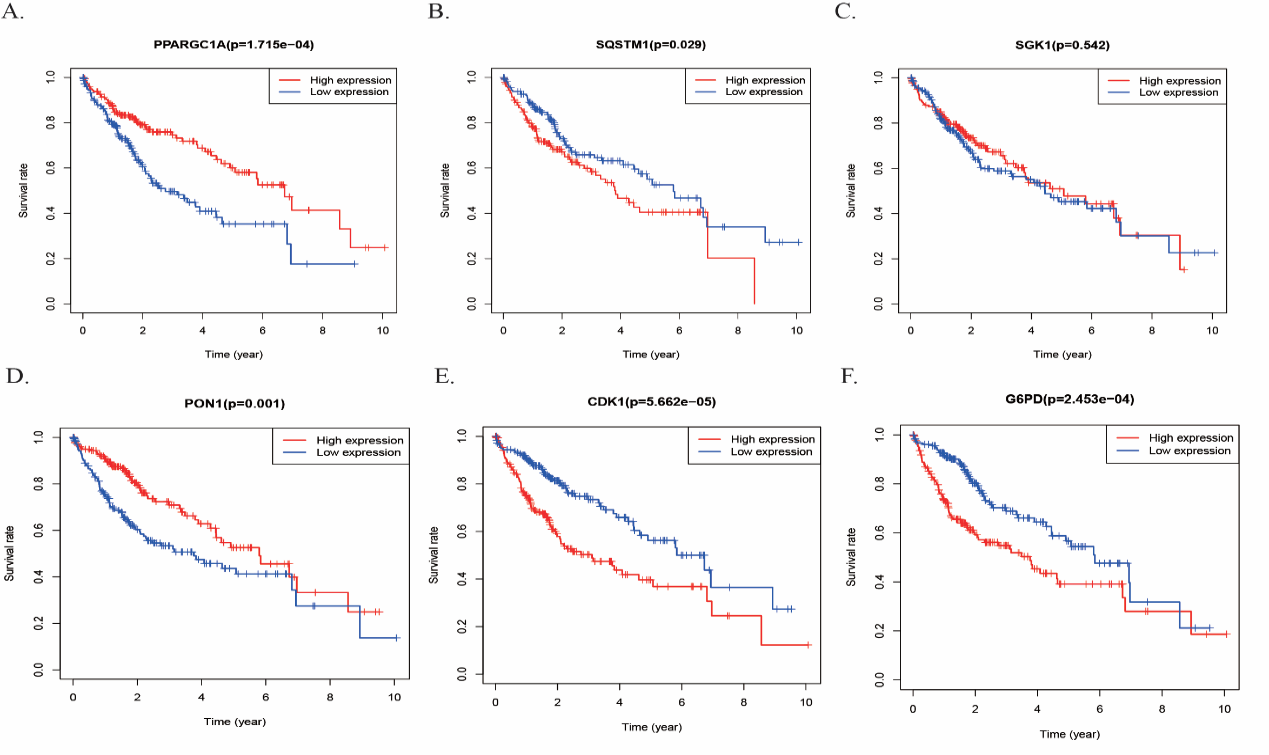


**Figure S4. The Kaplan-Meier survival curves based on the expression of six DE-ERGs with HCC in the TCGA-LIHC cohort.**


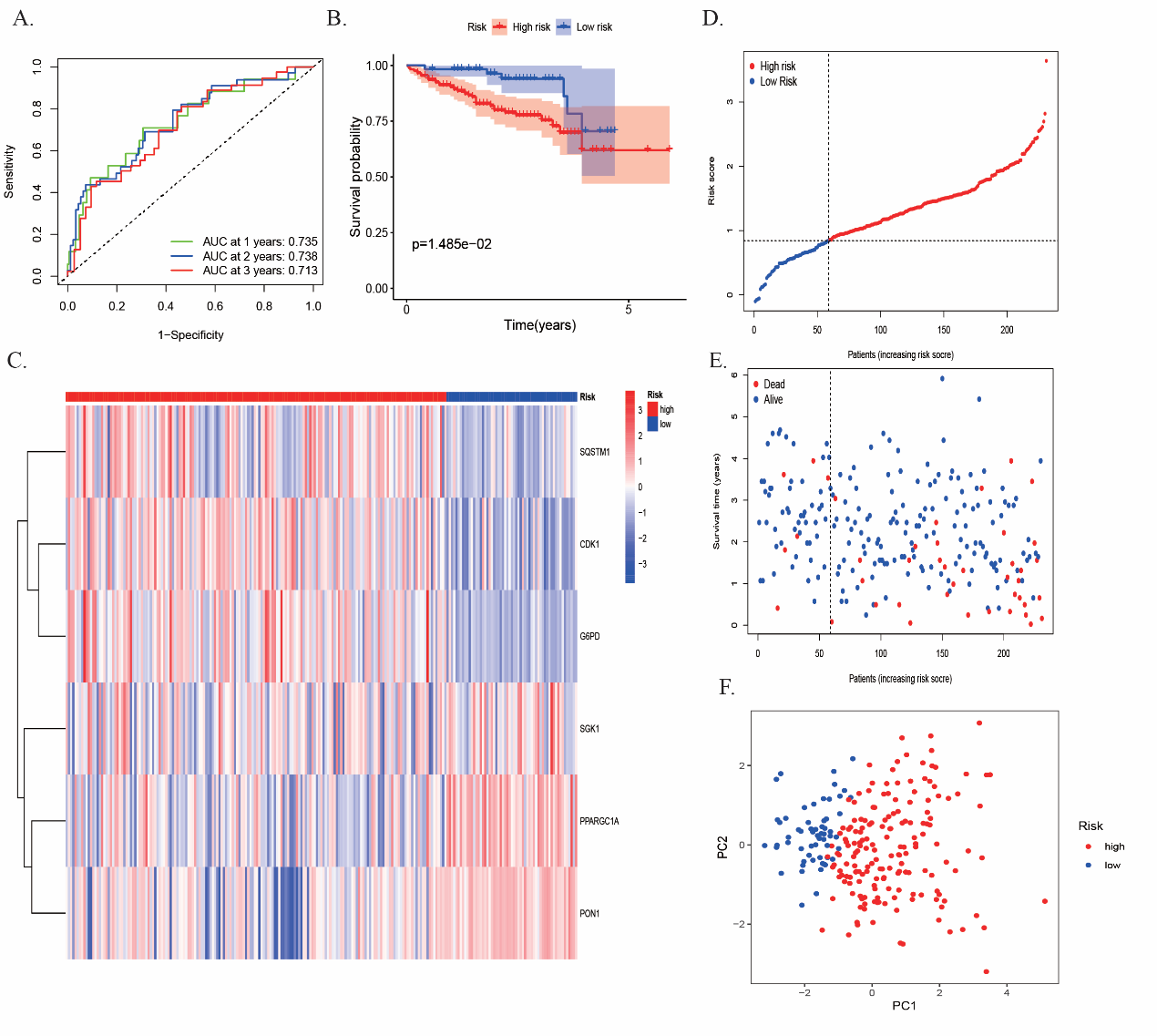


**Figure S5.** **KM survival analysis, risk score assessment by the ERG-related gene signature and time-dependent ROC curves in the ICGC cohort.** (A) ROC curve for overall survival of the validation set. The AUC was assessed at 1, 2, and 3 years. (B) KM survival analysis of high-and low-risk samples. (C) Six ERGs expression patterns for patients in high- and low-risk groups by the six ERG signature. (D) and (E). Relationship between the risk score rank/ survival status and risk score rank/ survival time (days). (F). PCA analysis for HCC patients.


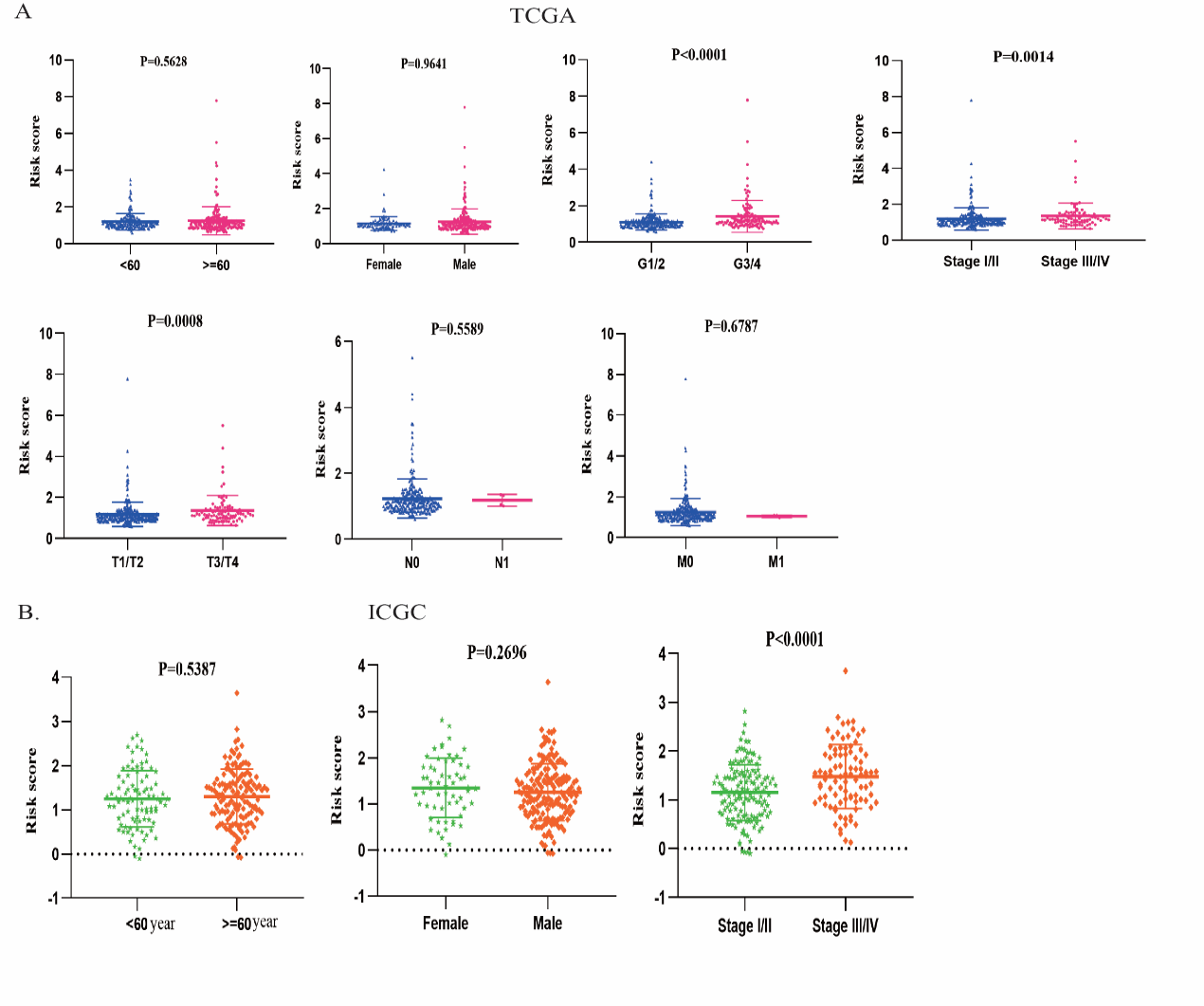


**Figure S6. The relationships between the ERGs related risk signature and (A)age, gender, grade, AJCC stage, T stage, N stage and M stage in TCGA-LIHC cohort; and (B) age, gender, and clinical stage in ICGC-LIRI-JP.**


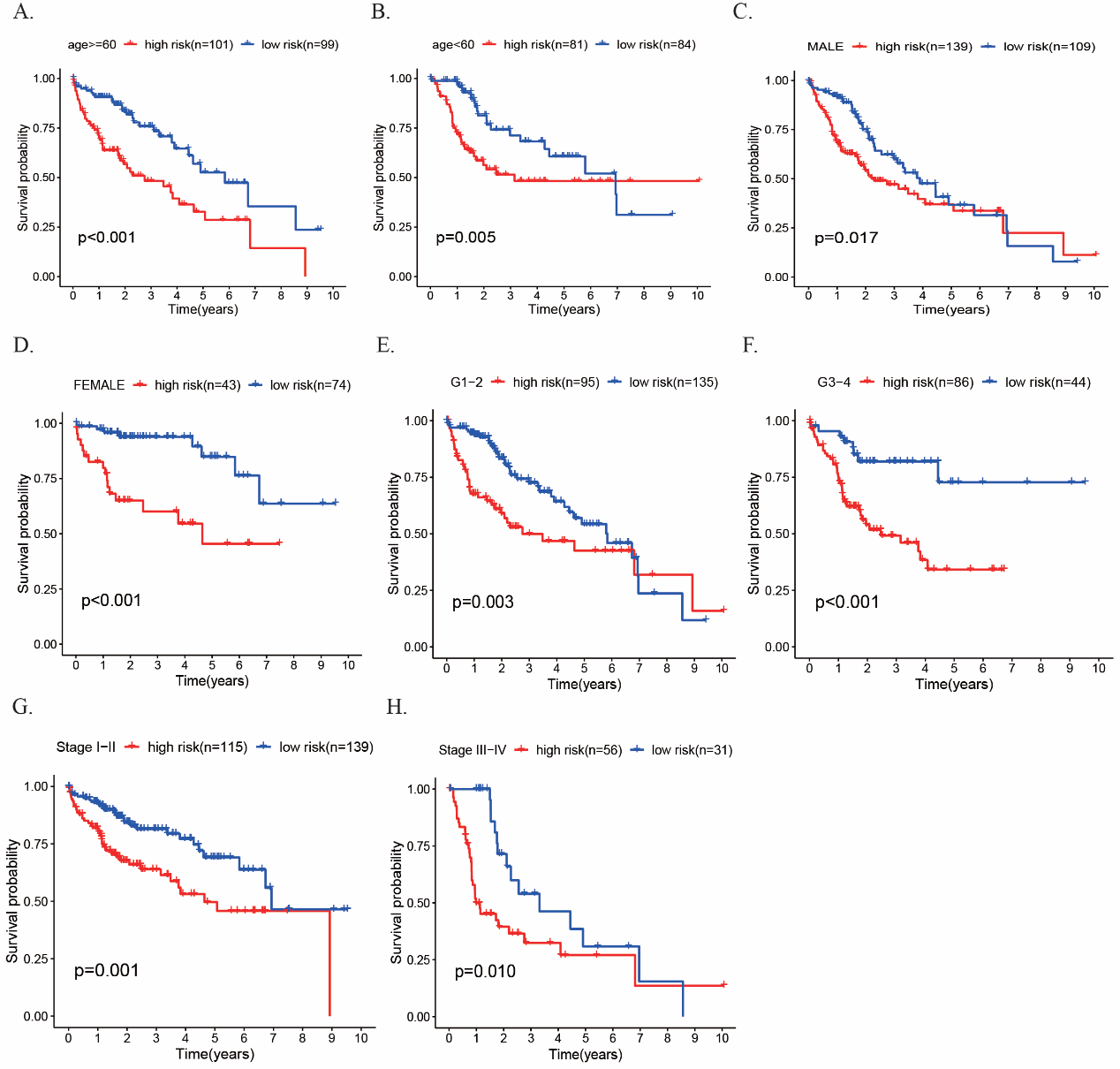


**Figure S7. Stratification analysis of various clinical features by Kaplan-Meier survival curves for the patients with CRC in the TCGA-LIHC cohort.** A-B. age; C-D. gender; E-F. T stage; G-H. N stage; I-J.M stage; K-L. AJCC stage.


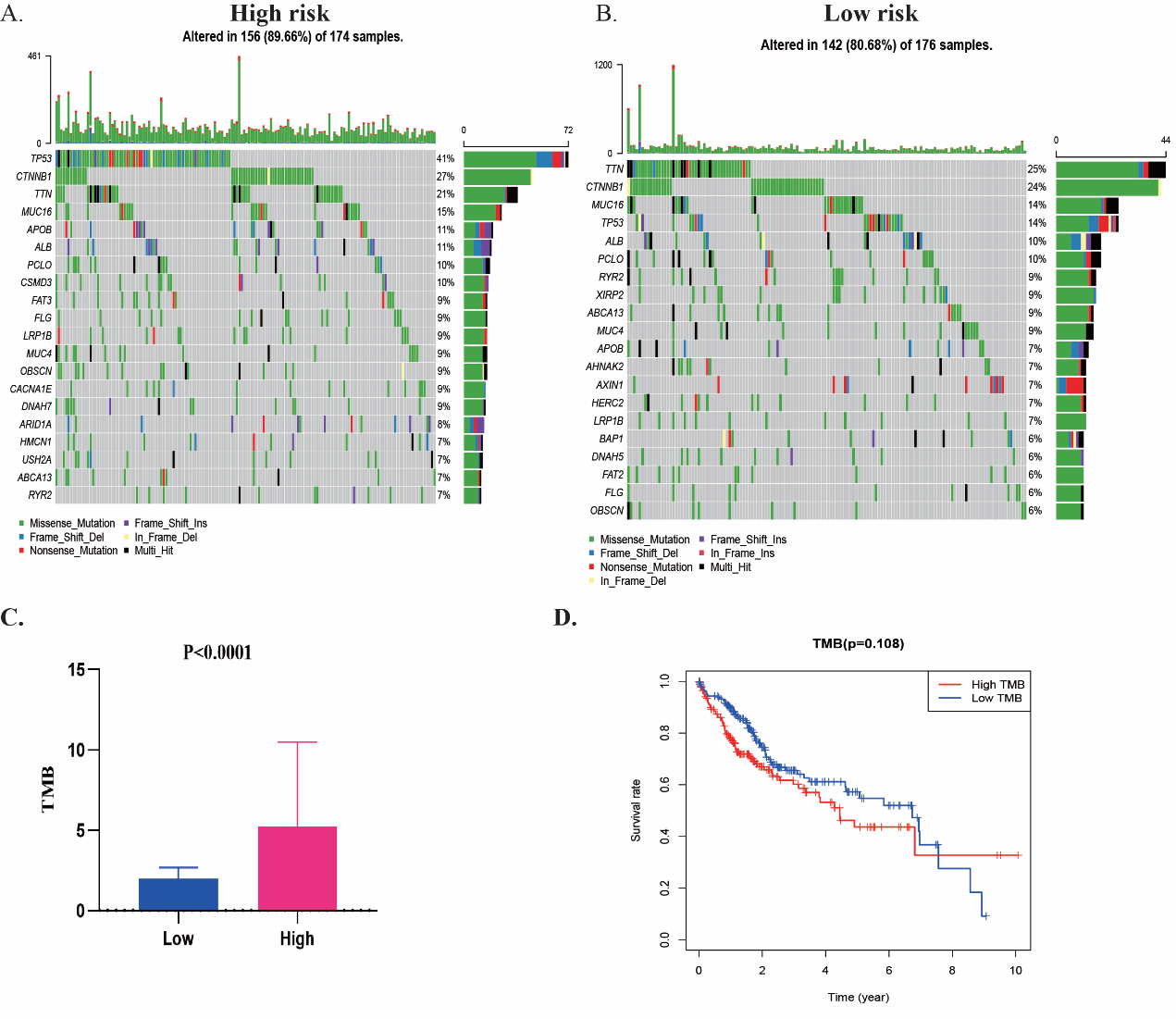


**Figure S8. The mutation profile and TMB among Low-risk and High-risk groups.** The top 20 most frequently mutated genes’ mutation profile in (A) High-risk and (B) Low-risk groups. (C) The relationship between the immune related risk signature and TMB. (D) The association of TMB and OS in the TCGA-LIHC cohort dataset.


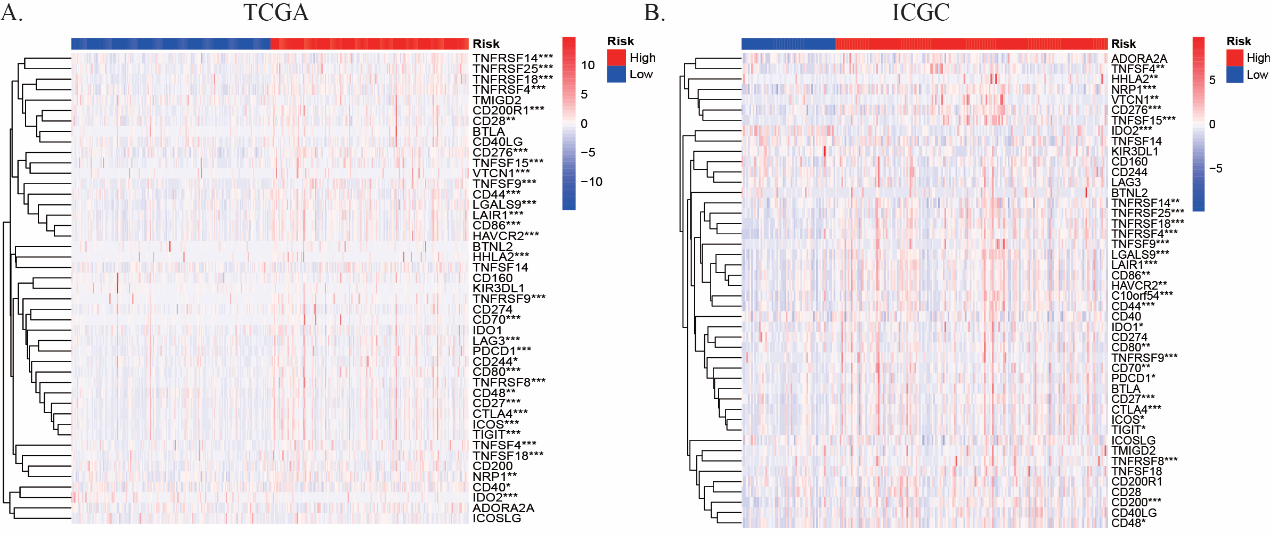


**Figure S9. The heatmap of immune-checkpoint expression in TCGA-LIHC cohort (A) and ICGC-LIRI-JP(B).**


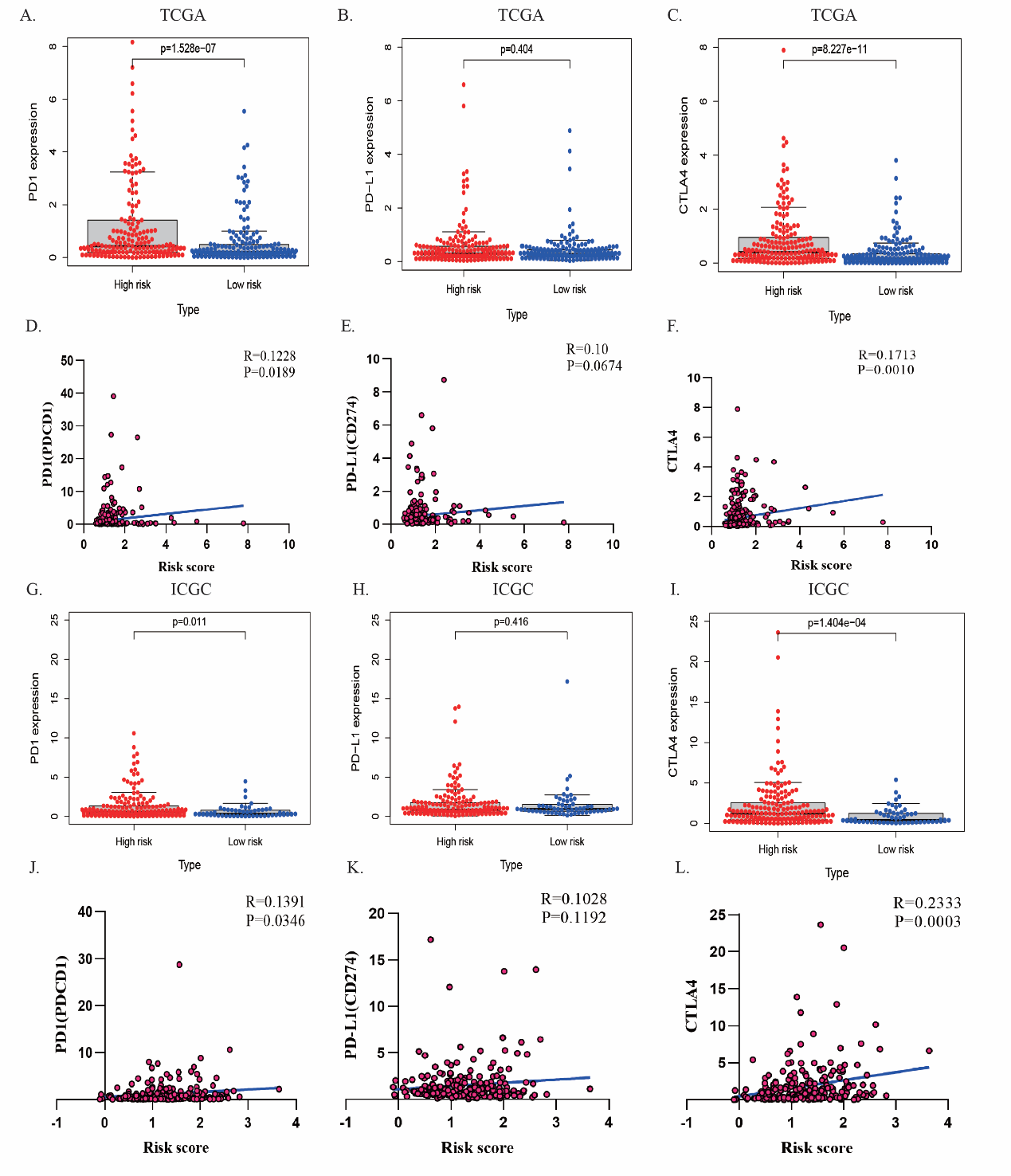


**Figure S10. Immune-checkpoint gene expression analysis in risk model.** (A-C) The gene expression of PD1, PD-L1 and CTLA4 in low-risk and high- risk groups in the TCGA-LIHC cohort. (D-F) The association between immune-checkpoint and the ERG-related risk signature in the TCGA-LIHC cohort. (G-I) The gene expression of PD1, PD-L1 and CTLA4 in low-risk and high- risk groups in the ICGC cohort. (J-L) The association between immune-checkpoint and the ERG-related risk signature in the ICGA cohort.
